# Microplastic Transport in Buckwheat Root-Inspired Microfluidic Structures: Microfluidic and Numerical Analysis

**DOI:** 10.3390/plants15081211

**Published:** 2026-04-15

**Authors:** Skaistė Dreskinienė, Monika Vilkienė, Gintarė Šidlauskaitė, Julija Pupeikė, Vykintė Trakšelytė, Paulius Vilkinis, Aistė Tilvikaitė, Justas Šereika

**Affiliations:** 1 Lithuanian Research Centre for Agriculture and Forestry, Instituto al.1, LT-58344 Akademija, Lithuania; monika.vilkiene@lammc.lt (M.V.); gintare.sidlauskaite@lammc.lt (G.Š.); 2 Center for Physical Sciences and Technology, Department of Textiles Technology, Demokratų Str. 53, LT-48485 Kaunas, Lithuania; vykinte.trakselyte@ftmc.lt; 3 Laboratory of Heat-Equipment Research and Testing, Lithuanian Energy Institute, Breslaujos Str. 3, LT-44403 Kaunas, Lithuania; aiste.tilvikaite@ktu.edu (A.T.); justas.sereika@lei.lt (J.Š.)

**Keywords:** microplastics, root epidermis, migration pathways, polyester microfibers, microfluidic modelling

## Abstract

Microplastics released from synthetic textiles are increasingly recognized as an important source of environmental contamination and a potential pathway of their entry into soil–plant systems. This study quantified microfibre release from warp-knitted polyester fabric during domestic washing and investigated the migration behaviour of microplastics within root epidermis-like structures using a combined experimental and numerical approach. Microfibre emission was determined gravimetrically according to ISO 4484-1:2023. The average release per washing cycle was 0.6 ± 0.5 g of microfibres per kilogram of polyester textile. Raman spectroscopy and differential scanning calorimetry analysis confirmed that the released particles consisted of polyethylene terephthalate. Scanning electron microscopy of buckwheat (*Fagopyrum esculentum*) roots revealed a well-defined epidermal and cortical tissue organization, which served as a basis for designing simplified epidermis-inspired microchannel geometries. Numerical simulations and microfluidic experiments showed that microplastics predominantly follow streamline-oriented pathways under laminar flow conditions. However, particle accumulation can induce localized clogging within pore-like structures, modifying flow pathways and redirecting particle transport. These results indicate that root epidermal tissues may function as a partial filtration barrier that restricts the transport of larger microplastics while allowing smaller particles to migrate through outer root layers.

## 1. Introduction

Microplastics (MPs) are anthropogenic particles smaller than 5 mm that have emerged as a global environmental concern [1] due to their persistence, ubiquity, and potential biological impact [2]. MPs occur in diverse shapes [3], including spheres, fibers, fragments, films, and pellets, with particle morphology playing a critical role in their environmental behaviors and biological interactions. Approximately 35% of MPs in the environment originate from synthetic textiles [4], and an estimated 13,000 tons of MPs are released annually in the EU due to the washing of synthetic fabrics [5].

Soil ecosystems represent an important sink for MPs [6], with agricultural soils acting as major accumulation reservoirs. Fibrous MPs, frequently reported as one of the dominant MP forms, enter agricultural systems primarily through two pathways: indirectly via treated wastewater reused [7] for irrigation or discharged into surface waters, and directly through the land application of sewage sludge as fertilizer [8]. Once introduced into soil, MPs interact with soil structures, pore networks, and biota, influencing their mobility and persistence [9]. The morphological characteristics of MPs, including particle size, shape, and polymer type [10], strongly influence their mobility through soil pore networks and plant tissues, thereby affecting their environmental distribution and associated ecological risks. For instance, spherical MPs tend to migrate more readily through soil pores than fibrous or fragmented particles [11] due to reduced entanglement and blockage potential, whereas fibrous MPs often accumulate within soil matrices and root-associated zones.

In recent years, research on MPs has expanded rapidly [12]; however, most studies remain focused on quantifying MP abundance, distribution [13,14], and physicochemical properties in environmental matrices such as soil [15] and air [16]. Comparatively less attention has been devoted to understanding MP uptake, internal transport, and accumulation within plants, despite increasing evidence that MPs are bioavailable to a wide range of organisms and may reach humans through trophic transfer along the food chain [17,18]. Experimental studies indicate that MPs can accumulate in the rhizosphere and root tissues [19,20], where they interfere with nutrient uptake, alter root morphology, and affect plant growth and physiological performance [21].

Exposure to MPs has been shown in experimental systems to induce oxidative stress [22], genotoxic effects [23], cytological alterations [24], and disruption of key physiological processes in plants, including photosynthesis and nutrient assimilation [25]. In addition, MPs may modulate gene expression, influence plant defense responses, and act as vectors for heavy metals and pathogenic microorganisms, potentially amplifying their ecological and agricultural impacts [26]. Nevertheless, detailed understanding of MP migration pathways within plants, particularly transport from roots to shoots and edible tissues, remains limited, largely due to methodological challenges associated with tracking small particles in complex biological matrices.

Buckwheat (*Fagopyrum esculentum*) represents a suitable model plant for investigating MP–plant interactions due to its well-developed root system, rapid growth, and known physiological plasticity under environmental stress [27].

Previous studies indicate that buckwheat roots exhibit active biochemical and molecular responses to MP exposure, including changes in stress-related metabolism and antioxidant activity, suggesting a potential adaptive or detoxification response [28]. However, the mechanisms governing MPs migration through buckwheat roots and their potential translocation within the plant remain poorly understood.

Innovative experimental approaches are required to overcome current limitations in MP transport research [29]. Microfluidic technologies offer unique opportunities to mimic plant vascular systems under controlled laboratory conditions, enabling visualization and quantitative analysis of particle transport processes that are otherwise difficult to observe in intact plants [30]. Microfluidic devices have been successfully applied to replicate physiological features of plant tissues and to simulate fluid flow dynamics, providing new insights into particle migration, retention, and accumulation at microscale resolutions.

We hypothesize that microfibres released from polyester (PES) textiles during domestic washing can enter plant systems, where the root epidermal structures of buckwheat act as a partial filtration barrier that modulates MP transport by promoting particle accumulation and localized clogging within pore-like pathways. The aim of this study was to quantify microfibre release from PES textiles during domestic washing and to investigate the transport behaviour of MPs within root epidermis-like structures based on the root morphology of buckwheat using a combined experimental and numerical approach.

## 2. Materials and Methods

### 2.1. Plants Growth Conditions

Buckwheat plants (variety ‘VB Nojai’, *Fagopyrum esculentum* Moench) were cultivated in a field experiment conducted within the international project Baltic Phytoremediation (BAPR) at the Vėžaičiai experimental site (Klaipėda district, Lithuania; 55°43′ N, 21°27′ E). The site is located on a morainic plain of the Eastern Žemaičiai Upland at an elevation of 95–100 m above sea level. The soil was classified as a typical unsaturated Retisol (Dystric Bathygleyic Glossic Retisol) with a morainic loam texture (clay content 13–15%).

The plants were sown approximately 90 days prior to sampling and harvested at the end of August. As the experiment was conducted under open field conditions, no artificial irrigation was applied, and plants were grown under natural environmental conditions.

A total of 40 buckwheat plants were randomly collected from the field for analysis. Root samples were obtained from each plant. Plant roots and stems were manually sectioned to obtain transverse cuts and mounted on 12 mm carbon adhesive tabs attached to stubs (Agar Scientific, Stansted, UK). The samples were air-dried for 24 h without conductive coating. Morphological and elemental analyses were performed using a FEI Quanta 650F scanning electron microscope (SEM) (FEI Company, Hillsboro, OR, USA) under high-vacuum conditions at an accelerating voltage of 5.0 kV.

### 2.2. MPs Preparation

**Figure 1:**
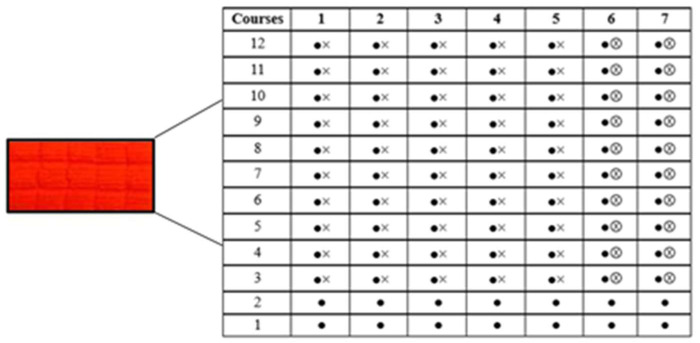
Plush stitch warp-knitted PES fabric sample along with its stitch layout (rapport) used in this study. The diagram represents 12 courses (rows) and 7 wales (columns). Filled circles (●) indicate ground stitches forming the base structure, crosses (×) denote loop intermeshing/binding points, and circled crosses (⊗) represent plush (pile) loops that are not integrated into the ground structure. Free pile loops are formed predominantly in the sixth and seventh wales, contributing to the raised surface morphology of the fabric.

A 100% dyed warp-knitted PES fabric sample was selected for this study (Figure 1). PES fabrics with plush structures are reported to release relatively higher amounts of microfibres during laundering due to the presence of raised pile loops and surface fibers that are more susceptible to mechanical stress [31,32]. Therefore, this fabric type was chosen as a representative structure with an increased potential for MP release.

The fabric was manufactured by Omniteksas, UAB (Kaunas, Lithuania). An intensely coloured fabric was deliberately selected to facilitate the visual identification and quantification of released microfibres collected on the filter medium during washing experiments.

The main technical characteristics of the investigated fabric are presented in Table 1. The fabric was manufactured on a single-needle bar warp knitting machine (gauge 20E). The stitch type corresponds to plush stitch (3.1.16 according to ISO 8388:1998) [33]. The ground structure was formed using PES textured yarns of 11.1 tex with 96 filaments, ensuring structural stability and dimensional integrity. The pile structure was formed using coarser PES textured yarns of 16.7 tex with 288 filaments, contributing to the formation of raised loops on the fabric surface.

The structural repeat consists of 12 courses and 7 wales. The first two courses form the ground structure, which provides the base framework of the fabric. Plush stitches are introduced from the fourth course onwards, gradually developing the pile effect. Free pile loops, which are not lapped into the ground structure, are formed exclusively in the sixth and seventh wales.

### 2.3. Raman Spectroscopy

After washing, the wash water was collected in full for subsequent filtration and analysis of released MPs fibers. Water with MPs was vacuum filtered through a metal filter (0.025 mm diameter) and air-dried. The polymer composition of the collected MPs was verified by Raman spectroscopy using a Raman Station 400F spectrometer (PerkinElmer, Waltham, MA, USA) equipped with a 785 nm excitation laser and a CCD detector. The 785 nm laser wavelength was selected to minimise fluorescence effects commonly observed in dyed textile fibres.

### 2.4. Differential Scanning Calorimetry (DSC)

Differential scanning calorimetry (DSC) analysis was performed using a DSC Q10 differential scanning calorimeter (TA Instruments, New Castle, DE, USA). Approximately around 4 mg of sample was sealed in an aluminum pan and analyzed under a nitrogen atmosphere with a purge flow of 50 mL min^−1^. A heat–cool–heat program was applied: heating from 35 °C to 280 °C at 35 °C min^−1^, cooling to 35 °C at 10 °C min^−1^, followed by a second heating to 280 °C at 35 °C min^−1^. Thermal parameters were determined from the second heating cycle.

### 2.5. Determination of Material Loss from Knitted Fabrics During Washing

After manufacturing, the fabric was pre-washed to remove manufacturing residues, finishing agents, and loosely attached fibres, thereby preventing potential interference with the experimental results. Washing was performed according to ISO 6330:2021 [34], procedure 4N (domestic washing at 40 °C) using a reference detergent without optical brightener and with a ballast load. The rinsing cycle was repeated twice following the washing procedure but without detergent. The fabric was then laid flat to dry. After pre-washing, the MP collection procedure was performed under controlled laboratory conditions, as described in the following section.

The release of MPs from knitted textile materials during laundering was determined in accordance with ISO 4484-1:2023 [35], Textiles and textile products—MPs from textile sources—Part 1: Determination of material loss from fabrics during washing. A total of eight rectangular specimens were prepared from the warp-knitted PES fabric. Four specimens were cut in the wale direction and four in the course direction to evaluate potential structural anisotropy in microfibre release. Initially, specimens measuring (150 ± 10) mm × (290 ± 10) mm were cut. Each edge of the specimen was folded twice towards the reverse side to form a double rolled hem (approximately 10 mm wide), using a total seam allowance of 25 mm. The hems were sewn using a single-needle lock stitch (type 301) with PES sewing thread. After hemming, the final specimen dimensions were (100 ± 10) mm × (240 ± 10) mm. Hemming was applied to prevent excessive fibre loss from cut edges and to ensure that the measured microfibre release originated predominantly from the fabric surface rather than from mechanically damaged specimen boundaries. The washing procedure was performed in accordance with ISO 4484-1:2023. Each washing experiment was repeated four times to ensure reproducibility of the results. Microfibre release was quantified gravimetrically after each washing cycle. The results are reported as mean values with standard deviation (mean ± SD).

Specimens were dried in a FD-53 laboratory oven (Binder GmbH, Tuttlingen, Germany) at 50 ± 3 °C until constant mass was achieved—for 4 h. After drying, specimens were cooled in a desiccator for 1 h and weighed using a KERN ACJ 200-4M analytical balance (KERN & Sohn GmbH, Balingen, Germany) with a precision of 0.0001 g.

Glass fibre filters (0.7 µm pore size, 47 mm diameter, binder-free) from Macherey-Nagel, Germany, were used for fibre collection. Each filter was dried at 50 ± 3 °C for 4 h and cooled in a desiccator for 1 h. Initial mass of knitted textile specimens and filters were recorded after cooling.

Washing was carried out using a laboratory SCOUROTESTER, FE-09/A laundering device (SDL Atlas, Rock Hill, SC, USA) consisting of stainless-steel canisters mounted horizontally in a thermostatically controlled rotating water bath. For each specimen, 250 mL of distilled water and 10 stainless steel balls were added to a stainless-steel canister and preheated in the laundering machine to 40 ± 3 °C temperature. One specimen was added per canister. The canisters were rotated at 40 ± 2 rpm for 45 ± 1 min at 40 ± 3 °C temperature. In accordance with ISO 4484-1:2023 standard Annex A, detergent was not used to avoid filter clogging and mass interference.

After washing, the entire wash liquid from each canister was filtered separately to prevent cross-contamination. Wash liquid was poured through a stainless-steel sieve to collect the specimen and stainless-steel balls. Everything (canister, specimens, steel ball, stainless steel sieve) was rinsed three times with distilled water and collected into a glass beaker.

All the collected water for each specimen separately was vacuum filtered through weighed glass filters. The beaker and filtration funnel were rinsed three times to ensure complete fibre transfer. Filters were then dried at 50 ± 3 °C for 4 h, cooled in a desiccator for 1 h, and weighed.

To avoid contamination all steps were done with recommendations of standard Annex A, A.2—Quality Control to Reduce Contamination.

To calculate the amount of microfibers released from the fabric, formulas from the standard ISO 4484-1:2023 were used:

The mass of fibre fragment release for each specimen:

Mf = Fm_2_ − Fm_1_ (1)

where:

Mf—mass of fibre released.

Fm_1_—mass in grams of filter paper before testing.

Fm_2_—mass in grams of filter paper after testing.

The percentage of fibre fragment release of each specimen:

(2) $$\mathrm{Pf}\;=\;\frac{Mf}{Sm_{1}}\;\times \;100$$

where:

Pf—percentage of fibre released.

Mf—mass of fibre released.

Sm_1_—mass in grams of specimen before testing.

Statistical analysis of the experimental data was performed using Microsoft Excel (Microsoft Corporation, Redmond, WA, USA). The results are expressed as mean values ± standard deviation (mean ± SD). To evaluate the effect of fabric cutting direction (wale and course) on microfibre release, an independent samples *t*-test was applied. This test was selected as appropriate for comparing the means of two independent groups. Statistical significance was determined at a 95% confidence level, with *p* < 0.05 considered statistically significant.

### 2.6. Numerical Simulations

Numerical simulations were performed using ANSYS Fluent 2019 R2 (ANSYS Inc., Canonsburg, PA, USA) in a two-dimensional domain. It represents root epidermis-inspired microchannel geometries with periodically arranged cylindrical obstacles. A pressure-based solver with absolute velocity formulation was employed. The simulations were conducted under transient conditions. The liquid phase was chosen to be water; therefore, it was an incompressible Newtonian fluid with constant properties: density *ρ*_f_ = 998.2 kg/m^3^ and dynamic viscosity *μ* = 0.001003 kg/(m·s). The flow regime was assumed laminar; therefore, no turbulence model was applied. A multiphase Eulerian framework coupled with the Dense Discrete Phase Model was used to account for particle transport. The DPM was activated with unsteady particle tracking. DPM source terms were updated at every flow iteration to ensure phase coupling. The particle time step was set to 10^−4^, with a maximum of 500 tracking steps per particle. MPs were simulated as inert spherical particles with a density of *ρ*_p_ = 1350 kg/m^3^ and diameter 5·10^−6^ m. Particle motion was solved in a Lagrangian framework according to Newton’s second law. At the inlet, a uniform velocity boundary condition was imposed for the water phase, while the outlet was defined as a pressure outlet with zero-gauge pressure. No-slip boundary conditions were applied on all solid surfaces. Particle injection was defined as a single injection from the inlet centerline with initial velocity components. The injection time interval was set from 0 to 0.1 s. Transient simulations were performed using a fixed time-stepping scheme with Δt = 10^−4^ s and a total of 50,000 timesteps. A maximum of 20 iterations per time step was allowed to ensure convergence within each transient increment.

### 2.7. Experimental Measurements

Microchannels were produced using a soft lithography technique. The microchannels were fabricated by first creating a master mold using SU-8 photoresist patterned on a silicon wafer through photolithography. A total of 50 g of liquid PDMS (10:1 polymeric base to curing agent) was then poured over the mold and heat-cured at 80 °C for 2 h. Once solidified, the PDMS layer was peeled off, revealing the embossed microchannel structure. Then PDMS layer and glass slides were exposed to 0.5 Torr air plasma for 2 min. Finally, a PDMS layer is plasma-bonded to glass to form sealed microchannels.

Polystyrene spherical microparticles (Merck Group, Darmstadt, Germany) with diameters of 5, 10, and 20 µm were used as a substitute for MPs. These particles were selected to isolate hydrodynamic transport mechanisms under controlled conditions. It is known that they do not fully represent the shape and surface properties of fibrous MPs, which may influence transport behavior in natural systems. The microparticles were suspended in distilled water containing 1% (*v*/*v*) Tween 20 to reduce particle aggregation and adhesion to the channel walls. Particle concentration was kept low to maintain single particle tracking conditions, approximately 10^3^–10^4^ particles/mL, which corresponds to ~6 × 10^−4^ to ~4 × 10^−2^% *v*/*v* for 5 µm and 20 µm particles, respectively. A syringe pump was used to maintain a stable flow rate in the microfluidic chip.

Spherical beads were chosen for the initial experiments as a simplified model system, enabling a clear and reproducible evaluation of migration. By using particles with a uniform geometry, the influence of size and transport mechanisms could be assessed without the added complexity introduced by shape-related effects. Future studies will extend this approach to MPs with different morphologies, including fibers and irregular fragments, in order to obtain a clearer and more comprehensive picture of MP migration in the plant system.

Clogging was achieved by allowing 20 µm microbeads to flow through the channel and waiting for some of them to become trapped between the pillars. Therefore, the clogging process was fully stochastic. Particle trajectories were obtained after the flow in the channel had stabilized. A constant flow rate was maintained for approximately 20 min before the measurements.

## 3. Results

### 3.1. Microfibre Release During Washing

Microfibre release from the warp-knitted PES fabric was quantified gravimetrically following domestic washing in accordance with ISO 4484-1:2023.

A total of eight specimens were prepared: four cuts in the wale direction and four in the course direction. Each specimen was subjected to four repeated washing cycles under identical conditions, resulting in a total of 32 measurements (*n* = 32). The results are presented as mean values with standard deviation (mean ± SD).

The mean percentage of fibre fragment release per washing cycle was:

0.06 ± 0.05% of the initial fabric mass (*n* = 32).

When normalised per unit mass of textile, this corresponds to:

0.60 ± 0.50 g of microfibres per kilogram of PES fabric per washing cycle.

Although some variability between measurements was observed, no statistically significant difference in microfibre release was detected between specimens cut in the wale and course directions.

**Figure 2:**
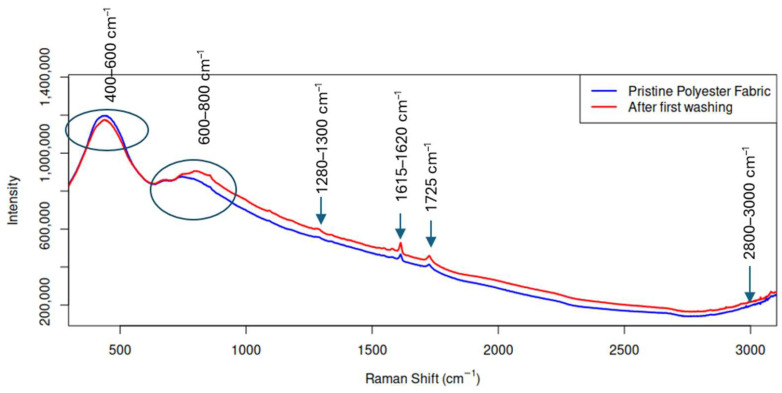
The Raman spectra of pristine PES fabric and MPs collected after the first washing cycle.

Microfibres were retained using 0.7 µm pore size glass fibre filters, ensuring capture of micrometre sized PES fragments. Raman spectroscopic analysis confirmed (Figure 2) that the released particles correspond to polyethylene terephthalate (PET), consistent with the composition of the investigated textile [36].

### 3.2. Textile Analysis

Raman spectra were collected in the range of 200–3200 cm^−1^ with a spectral resolution of approximately 4 cm^−1^, Figure 2. Multiple spectra were acquired from different measurement points on each sample to ensure representativeness. Background subtraction and baseline correction were applied prior to spectral analysis. The resulting spectra were compared with reference spectra from established polymer libraries and literature data to confirm polymer identification.

**Figure 3:**
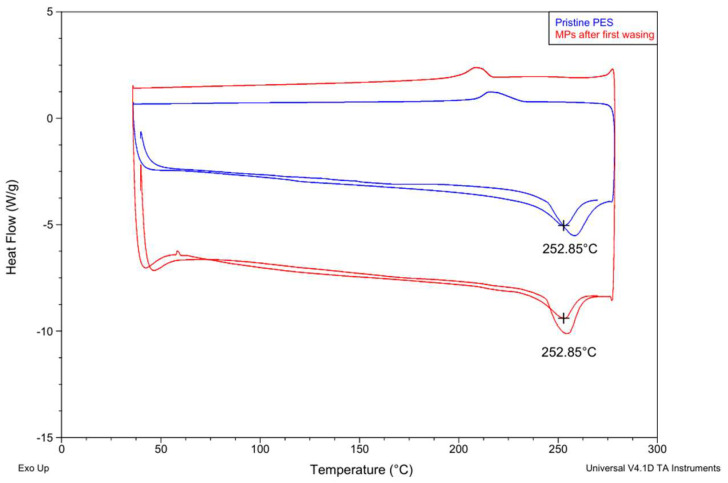
DSC thermograms of pristine PES fabric and MPs collected after the first washing cycle. The “+” symbol indicates the peak position corresponding to the characteristic thermal transition (melting temperature) of the material.

The Raman spectrum of the collected particles exhibits the characteristic vibrational bands of PET, consistent with published spectral assignments [37,38]. A dominant band at ~1725 cm^−1^ corresponds to the ester carbonyl (C=O) stretching mode, which is a hallmark feature of PET polymers [39]. Additional characteristic bands were observed in the ~2800–3000 cm^−1^ region attributed to C–H stretching vibrations, ~1615–1620 cm^−1^ assigned to aromatic ring C=C stretching, ~1280–1300 cm^−1^ associated with C–O–O stretching, and in the ~600–800 cm^−1^ region corresponding to aromatic ring deformation and C–H bending vibrations [40,41,42] (Figure 3). These spectral features closely match reference spectra reported in previous studies on PES textiles and MPs identification.

A comparison with spectra from pristine PES fabric indicates that the spectral positions of the main bands remain consistent with PET, confirming that the collected particles originate from the knitted PES sample. The relative intensities of the Raman bands were qualitatively higher in the washed samples, which may be attributed to an increased number of MPs within the laser sampling area after laundering. It should be noted that Raman signal intensity is dependent on particle concentration, orientation, and surface morphology within the sampling volume; therefore, intensity differences were interpreted semi-quantitatively rather than as absolute concentration values [43,44].

The observed spectral features and their correspondence with established PES signatures support the conclusion that MPs released during domestic washing are composed of PET, in agreement with findings from recent textile MP release studies [45,46].

The DSC thermograms of the pristine PES fabric and the MPs collected after the first washing cycle show very similar thermal behavior. Both samples exhibit a distinct melting endotherm at approximately 252.85 °C, which is characteristic of PET, the polymer composing PES fibers (Figure 3) [47,48,49]. The presence of the melting peak at the same temperature for both samples indicates that the collected particles originate from the PES fabric and retain the same polymeric structure.

Minor differences in the peak shape and intensity can be observed between the pristine material and the washed sample. The MP sample shows a slightly more pronounced melting peak, which may be associated with differences in particle size, morphology, or crystalline structure resulting from mechanical fragmentation during washing. In the lower temperature region, a weak baseline shift corresponding to the glass transition region typical for PET can also be observed.

### 3.3. Root Tissues Analysis

**Figure 4:**
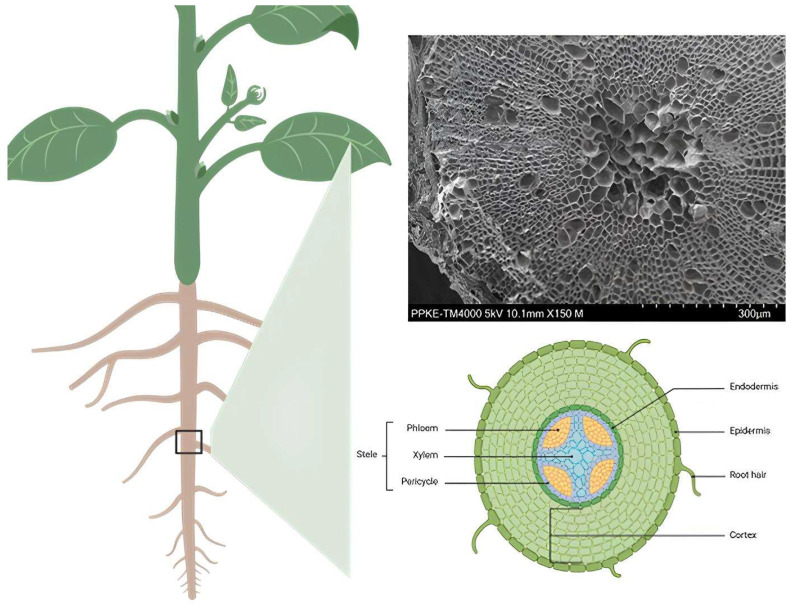
SEM image of transverse section cut of buckwheat root.

SEM micrographs of buckwheat root cross-sections revealed a well-defined tissue organization (Figure 4). The epidermis formed the outermost cell layer, followed by several layers of cortical parenchyma characterized by relatively large cell lumens. A distinct endodermal layer separated the cortex from the central vascular cylinder. Within the stele, xylem elements were located centrally, with phloem tissues positioned between them. Cell dimensions of the epidermal, cortical, and vascular tissues were quantified based on SEM images.

### 3.4. Numerical and Experimental Analysis of Fluid Flow and MP Trajectories Inside Root Epidermis-Inspired Channel

**Figure 5:**
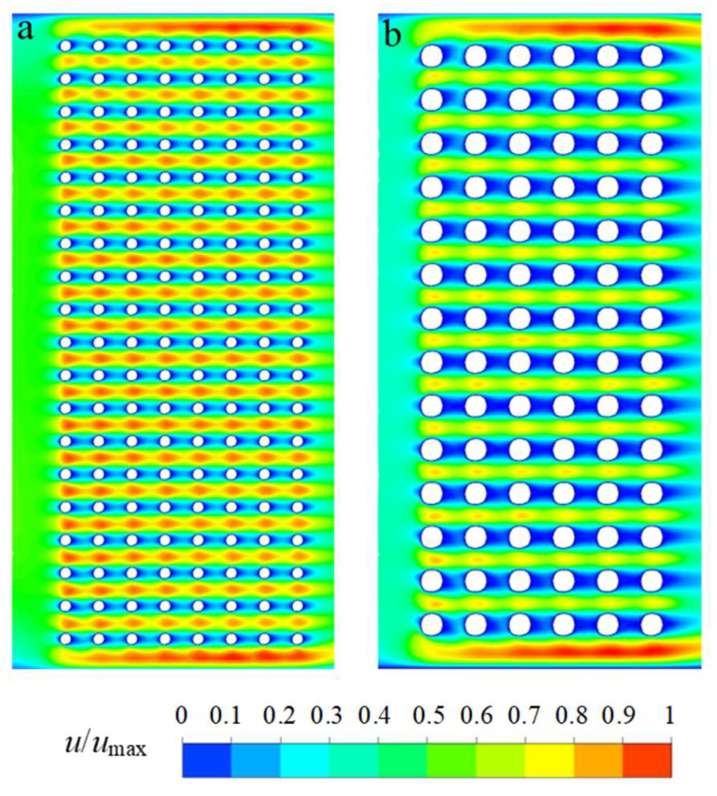
Normalized velocity magnitude distribution (u/umax) in epidermis-inspired microchannel structures; (**a**) pillar size 10 µm, gap size 20 µm; (**b**) pillar size 20 µm, gap size 20 µm.

Figure 5 presents the normalized velocity distribution (*u*/*u*_max_) within two microchannel geometries representing a simplified structure of the buckwheat root epidermis. Both domains contain periodically arranged two-dimensional cylindrical obstacles that mimic a porous medium. In both cases, the spacing between obstacles is 20 µm, while the obstacle diameters differ (10 µm and 20 µm). The numerically simulated velocity field is normalized by the maximum velocity in each case, enabling direct comparison of the resulting flow structures.

In Figure 5a, elongated high-velocity regions develop between adjacent rows of obstacles, indicating preferential flow pathways through the gaps between cylinders. The low-velocity regions downstream of the obstacles are short and weakly pronounced, remaining confined by the periodic obstacle arrangement. The resulting flow pattern resembles quasi-periodic porous-medium behaviour with localized micro-accelerations.

In Figure 5b, similar elongated high-velocity regions occur between obstacle rows, reflecting the identical spacing between obstacles (20 µm). The low-velocity zones behind individual obstacles remain short and confined to the immediate wake region. No clearly developed or extended recirculation structures are observed, indicating the dominance of viscous forces under laminar flow conditions.

**Figure 6:**
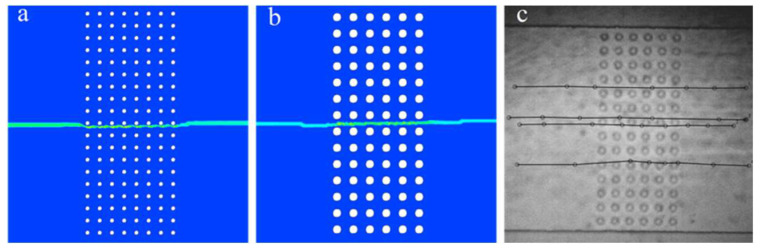
MP trajectories in epidermis-inspired microchannel geometry; (**a**) pillar size—10 µm, gap size 20 µm; (**b**,**c**) pillar size 20 µm, gap size 20 µm.

Figure 6 presents the computed trajectories of a 5 µm diameter MP transported through epidermis-inspired microchannel structures. The particle was introduced at the center of the inlet; therefore, the resulting trajectory remains approximately centered within the computational domain. In both cases (Figure 6a,b), the particle trajectory remains nearly linear across the structured region. Only minor lateral deviations occur as the particle passes successive rows of cylinders. This behavior is consistent with the relatively homogeneous velocity distribution characteristic of laminar flow. The particle closely follows the fluid streamlines, indicating that viscous forces dominate over inertial forces in this geometry.

In Figure 6, despite the increased obstacle diameter, the overall migration path remains similar and follows a streamline-oriented pattern across the channel. The particle moves through preferential flow pathways formed between obstacle rows, exhibiting only minor deviations as it passes successive constrictions. No trapping or significant recirculatory motion is observed for the investigated particle trajectory.

Figure 6c presents the experimentally measured trajectories of MPs–analog particles within the fabricated microfluidic structure. The experimental observations show good agreement with the numerical simulations, confirming the reliability of the numerical model.

**Figure 7:**
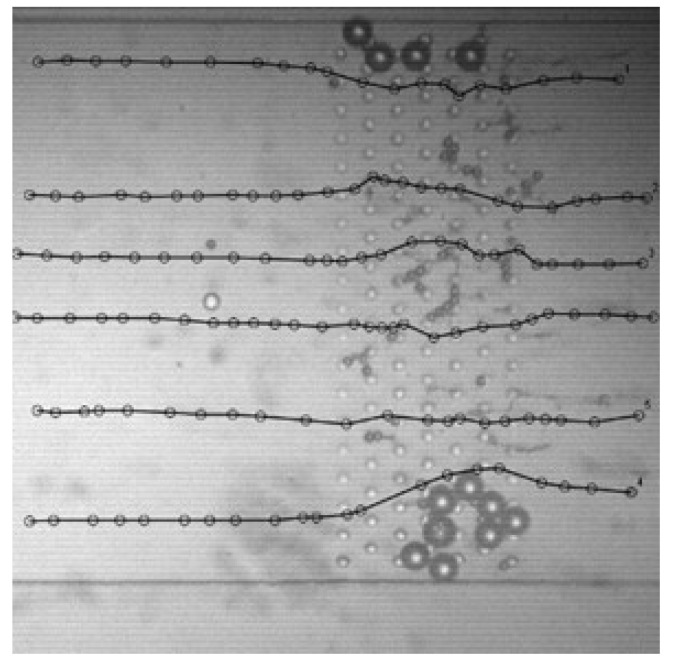
Experimentally observed particle trajectories in an epidermis-inspired microchannel during clogging. Stuck particle size—20 µm; flowing particle size—10 µm.

Figure 7 shows experimentally determined trajectories of particle flow under conditions of partial clogging. Clogging was intentionally induced by introducing particles with diameters of 5, 10, and 20 µm into the microchannel. Particles with sizes comparable to the characteristic gaps within the structure become trapped between the cylindrical obstacles, gradually forming localized blockages. As additional particles enter the system, accumulation occurs over time, progressively obstructing the flow pathways. This effect can be observed in regions where several 20 µm particles are trapped near the top and bottom of the structure, while smaller particles (5 µm) accumulate in the central region. The black lines represent trajectories of tracked particles obtained during the experiments. In contrast to the nearly linear trajectories observed in unobstructed structures, the presence of trapped particles significantly alters particle motion. Partial blockage redirects the flow, forcing incoming particles to deviate from their original paths and migrate through the least-resistant pathways remaining within the structure. Consequently, noticeable deflections and curvature appear in the trajectories as particles approach obstructed regions. This behavior demonstrates that particle accumulation can dynamically modify the effective permeability of the epidermis-like microstructure, thereby altering migration pathways of subsequently transported particles.

## 4. Discussion

Textile-derived microfibres released during washing may subsequently reach soils and interact with plant root systems. Here, textile washing experiments were combined with root-inspired microfluidic modelling to investigate both PES microfibre release and the potential transport behavior of MPs in epidermis-like structures.

Fibre release did not differ significantly between specimens cut in the wale and course directions, indicating that orientation had no effect on detachment under the tested washing conditions. The raised looped surface, which is less mechanically restrained than ground yarns [50], promotes detachment during laundering via bending, abrasion, and hydrodynamic forces [51], and the large SD likely reflects the plush stitch warp-knitted PES fabric’s structural heterogeneity, leading to uneven fibre release [52]. Repeated laundering during the lifetime of garments may result in substantial cumulative emissions of MPs entering wastewater systems [53]. A considerable fraction of these particles can subsequently accumulate in sewage sludge during wastewater treatment [54], which is frequently applied to agricultural soils as fertilizer [55]. As a result, textile-derived MPs can be transferred from domestic wastewater streams to terrestrial environments, where they can interact with soil structures and plant root systems.

Simulations and microfluidic experiments indicate that particle migration in epidermis-inspired structures is mainly controlled by laminar flow. Small particles (5 µm) moved along nearly linear trajectories with only minor deviations near constrictions. Such behavior is consistent with particle transport mechanisms observed in porous media and microfluidic systems [56], where viscous forces dominate at low Reynolds numbers [57]. These findings suggest that MPs may migrate through pore-like biological structures with relatively low resistance when particle size is much smaller than pore dimensions.

Experimental observations further showed that particles (≈20 µm) with diameters comparable to structural gaps can become trapped between obstacles, leading to localized clogging and altered trajectories. As particle accumulation increases, flow pathways are progressively redirected through the remaining open channels. Near obstructed regions, particle trajectories exhibit noticeable deflections and curvature, indicating that accumulation can dynamically modify the effective permeability of the epidermis-like microstructure and redirect the migration of subsequent particles.

These mechanisms are consistent with observations from plant–soil systems, where MP uptake is size-dependent. Nanoplastics (<1 µm) can penetrate root tissues and undergo internal transport [58], whereas larger MPs (≈1–10 µm) show reduced mobility and particles >10–20 µm are often retained within the outer root layers [59]. Consequently, the root epidermis may function as a filtration barrier that limits the passage of larger MPs while allowing smaller particles to migrate through the outer root layer with relatively low obstruction.

More broadly, MPs’ transport to plants is controlled by a combination of particle properties, soil characteristics, plant traits, and environmental processes. In addition to particle size, factors such as particle shape, polymer type, and surface charge affect interactions with soil particles and root tissues [60,61]. Charge-dependent adsorption of plastic particles to biological surfaces has been reported, highlighting the importance of electrostatic interactions [62,63,64]. Soil properties, including pore size distribution, texture, organic matter content, and pH, regulate particle mobility and retention within the soil matrix [65]. Plant-related factors such as epidermal structure, cell wall pore size, and root exudates may further influence MP transport in the rhizosphere [66]. Together, these factors determine whether MPs remain immobilized in soil or migrate toward plant root systems.

The combined experimental and numerical results suggest that MPs’ transport in plant–soil systems may depend strongly on particle size, pore geometry, and local flow conditions. Although the microfluidic model simplifies the structural complexity of natural soils and root tissues, it provides useful insights into fundamental particle transport mechanisms under controlled conditions. Microfluidic approaches therefore represent a promising tool for investigating particle transport mechanisms in complex biological structures and may contribute to improved understanding of MPs’ behaviour in agricultural environments.

## 5. Conclusions

This study quantified microfibre release from warp-knitted PES fabric during domestic washing and examined the migration behaviour of MPs in root epidermis-inspired structures using a combined experimental, microfluidic, and numerical approach. Gravimetric analysis revealed an average microfibre release of 0.06 ± 0.05% of the initial textile mass per washing cycle. Raman spectroscopy and DSC analysis confirmed that the released particles consisted of PET, and no statistically significant differences in fibre release were observed between specimens cut in the wale and course directions.

SEM analysis of buckwheat roots revealed a clearly defined epidermal and cortical tissue organization that served as the structural basis for simplified epidermis-inspired microchannel geometries. Numerical simulations demonstrated the formation of preferential high-velocity pathways between cylindrical obstacles under laminar flow conditions, while simulated particle trajectories followed predominantly streamline-oriented transport with minor lateral deviations.

Microfluidic experiments supported the numerical results and showed that particle accumulation can induce localized clogging when particle sizes approach the characteristic pore dimensions of the structure. Larger particles (≈20 µm) were more likely to become trapped between obstacles, whereas smaller particles (≈5 µm) migrated through the structure with limited obstruction.

These findings support the proposed hypothesis that the root epidermis acts as a size-selective filtration barrier, restricting the penetration of larger MPs while allowing smaller particles to migrate through outer root tissues. Overall, this study provides new mechanistic insights into MP transport at the soil–root interface and highlights potential pathways for their entry into plant systems.

Future work will focus on further investigating the MP transport mechanisms, while extending the current microfluidic model to incorporate biologically relevant features, such as heterogeneous pore structures and particle–tissue interactions.

## Figures and Tables

**Table 1:** Technical characteristics of the PES sample used in the study.

Parameter	Description
Knitting Machine	Single-needle bar warp knitting machine, gauge 20E
Stitch Type	Plush stitch (3.1.16 according to ISO 8388:1998)
Raw Material—Ground Yarns	PES textured yarns, 11.1 tex, 96 filaments
Raw Material—Pile Yarns	PES textured yarns, 16.7 tex, 288 filaments

## Data Availability

The original contributions presented in the study are included in the article, and further inquiries can be directed to the corresponding author.
